# Cytotoxic, Virucidal, and Antiviral Activity of South American Plant and Algae Extracts

**DOI:** 10.1100/2012/174837

**Published:** 2012-04-24

**Authors:** Paula Faral-Tello, Santiago Mirazo, Carmelo Dutra, Andrés Pérez, Lucía Geis-Asteggiante, Sandra Frabasile, Elina Koncke, Danilo Davyt, Lucía Cavallaro, Horacio Heinzen, Juan Arbiza

**Affiliations:** ^1^Sección Virología, Facultad de Ciencias, Universidad de la República, 11400 Montevideo, Uruguay; ^2^Cátedra de Productos Naturales Facultad de Química, Universidad de la República, 11800 Montevideo, Uruguay; ^3^Cátedra de Virología, Facultad de Farmacia y Bioquimica, Universidad de Buenos Aires, C1113AAD Buenos Aires, Argentina

## Abstract

Herpes simplex virus type 1 (HSV-1) infection has a prevalence of 70% in the human population. Treatment is based on acyclovir, valacyclovir, and foscarnet, three drugs that share the same mechanism of action and of which resistant strains have been isolated from patients. In this aspect, innovative drug therapies are required. Natural products offer unlimited opportunities for the discovery of antiviral compounds. In this study, 28 extracts corresponding to 24 plant species and 4 alga species were assayed *in vitro* to detect antiviral activity against HSV-1. Six of the methanolic extracts inactivated viral particles by direct interaction and 14 presented antiviral activity when incubated with cells already infected. Most interesting antiviral activity values obtained are those of *Limonium brasiliense, Psidium guajava,* and *Phyllanthus niruri*, which inhibit HSV-1 replication *in vitro* with 50% effective concentration (EC_50_) values of 185, 118, and 60 **μ**g/mL, respectively. For these extracts toxicity values were calculated and therefore selectivity indexes (SI) obtained. Further characterization of the bioactive components of antiviral plants will pave the way for the discovery of new compounds against HSV-1.

## 1. Introduction

Herpes simplex virus type 1 (HSV-1), a member of the Herpesviridae family, is a double-stranded DNA virus, extremely widespread in the human population [1, 2] with prevalence ranging from 60 to more than 95% [3]. It is responsible for a broad range of disorders, including gingivostomatitis, keratoconjunctivitis, genital disease, and encephalitis [4], causing more severe illnesses in newborns and immunocompromised patients [5].

Several drugs, acyclovir, vidarabine, phosphonoformic acid, and other compounds including interferon, are used to treat HSV-associated diseases [6]. However, an increasing amount of drug-resistant strains have been isolated in the last few years, thus limiting the efficacy of these drugs [7] and enhancing the need for drugs that innovate in the viral targets or/and the mechanisms of action.

It is estimated that 40% of modern drugs derived from natural sources [8]. Ethnopharmacological knowledge and texts of traditional herbal medicine usage have been an important source of information and have shown to be very efficient in the identification of bioactive compounds, even when compared to the standard high volume random screening method [9]. South American flora has been explored for bioactive compounds with remarkable results, including the antioxidant properties of *Achyrocline satureioides* commonly known as “Marcela” [10], capability of *Phyllanthus niruri* of dissolving kidney and gall stones [11], and the *Echinacea purpurea* anti-influenza virus properties [12].

Standardized cultivation of viruses is a powerful approach for *in vitro* drug testing that includes the possibility of comparison with reference drugs. The aim of this study was to challenge extracts of a set of South American plants to inhibit HSV-1 replication in a mammalian cell culture model. For this, 28 ethanolic extracts corresponding to 24 plant and 4 alga species were prepared and assayed *in vitro* to detect selective antiviral activity. All species are found in Uruguayan soil and reports describing usage in traditional medicine exist for all of them. Moreover, some of the plants are specifically recommended to treat genital, throat, eyes, and mouth sores and itching.

## 2. Materials and Methods

### 2.1. Plant and Algae Material

The collection of all plant and algae species was made in their natural environment in southern Uruguay, except for *Phyllanthus niruri *that was bought from Botica del Señor SRL (Lot N°3-Int. 1/07). The identification of all materials was performed by Lic. Alonso Paz and M. J. Bassagoda from the Department of Pharmaceutics Botanic of Facultad de Química, Universidad de la República. Voucher specimens have been deposited at the herbarium of Facultad de Química, Universidad de la República.

### 2.2. Preparation of Extracts

To prepare all ethanolic extracts the plant material was dried and macerated for 48 hours in EtOH : H_2_O (70 : 30 v/v). The algae material was air-dried and processed in the same way as plants except for *Grateloupia filicina*, where CHCL_3_ : MeOH (50 : 50 v/v) was used for maceration. The liquid fractions were freeze-dried and solubilized in DMSO to prepare stock and work solutions.

### 2.3. Cells, Virus, and Acyclovir

Vero cells (African green monkey kidney, ATCC number CCL-81) were cultured in D-MEM GlutaMAX-1 (Gibco Invitrogen) supplemented with 10% fetal bovine serum (FBS) (Gibco Invitrogen), 100 *μ*g/mL Streptomycin, and 1000 U/mL penicillin and grown at 37°C in a 5% CO_2_ humidified atmosphere. The cell line used in this work was *Mycoplasma* sp. free. HSV-1 strain F was provided by Dra. Lucía Cavallaro from Cátedra de Virología, Facultad de Farmacia y Bioquímica, Universidad de Buenos Aires, Argentina. Acyclovir was purchased from Laboratorio Libra (Virulax 250) and prepared to 1 mg/mL in sterile water.

### 2.4. Determination of MNCC and CC_50_

 To evaluate the effect of the extracts on Vero cells viability, dilutions ranging from 1000 to 16 *μ*g/mL were added on 85–95% confluent monolayers in 96-well culture plates. After 72 hours of incubation, the maximum noncytotoxic concentration (MNCC) for all extracts was determined by microscopic observation and CC_50_ (extract concentration that is toxic for half of the cells) was determined by the crystal violet uptake method. Briefly, cell monolayers were fixed and stained with a crystal violet 0.75% in 40% methanol solution and incubated for 15 minutes at 37°C. Nonincorporated crystal violet dye was removed. Retained dye in the cells was solubilized with an acetic acid 20% solution, and the absorbance at 570 nm was measured in a microplate reader (Bio-Rad, Model 680).

### 2.5. Screening of Antiviral Activity by Cytopathic Effect Reduction Assay

Primary antiviral screening test was conducted by cytopathic effect (CPE) reduction assay, which involves the protection of viral-caused lysis of Vero cells by extracts. Briefly, in 96-well culture plate, 5 × 10^4 ^UFP/mL of HSV-1 was inoculated on Vero cells. After 1 hour of adsorption at 37°C in a 5% CO_2_ humidified atmosphere, cells were washed with phosphate buffer saline (PBS) and 2-fold serial dilutions of the correspondent extract beginning at the MNCC were added. Cytotoxicity and cell controls were included. After 72 hours of incubation at 37°C in a 5% CO_2_ humidified atmosphere, cells were fixed and stained as described in Section 2.4. The antiherpetic activity of the extracts was evaluated by direct observation of CPE reduction. 

### 2.6. Determination of EC_50_ by Plaque Reduction Assay

 To determine the effective concentration that inhibited 50% of plaque forming units (PFUs) (EC_50_) a standard plaque reduction assay was conducted. Briefly, Vero cells monolayers grown in 48-well culture plates were infected with 100 PFU of HSV-1. After 1 hour of adsorption at 37°C in a 5% CO_2_ humidified atmosphere the cells were rinsed with PBS. Twofold serial dilutions of the correspondent extract (beginning from the MNCC) in medium containing 2% FBS and 0.8% methylcellulose were added. Each concentration was tested in duplicate. Infected cultures were incubated for 48 h at 37°C in a 5% CO_2_ humidified atmosphere. Cells were fixed and stained as described (see above). Plaques were counted and viral title expressed as PFU/well. Viral, cellular, and cytotoxic controls were performed in each assay. Acyclovir was included as a positive inhibition control.

### 2.7. Virucidal Activity Assay

To evaluate the presence of virucidal activity, direct inactivation of HSV-1 by the extracts was tested. To assay virucidal activity, a 5 × 10^9^ UFP/mL viral inoculum was mixed with extracts at three serial concentrations (MNCC, MNCC/2, and MNCC/4) and incubated at room temperature for 1 hour. After that, viral inoculum was diluted in medium supplemented with 2% FBS to a 500 UFP/mL concentration. Surviving infectious viral particles were measured by standard plaque reduction assay with respect to the control in which virus was mixed with medium supplemented with 2% FBS. Cell controls were included. Extract was considered positive when it inactivated ≥90% of the viral particles with respect to a control.

### 2.8. Data Analysis

CC_50_ and EC_50_ for each extract were obtained from dose-effect curves (data not shown). The values correspond to the average and standard deviation of three independent assays with at least 5 concentrations within the inhibitory range. The selectivity index (IS) is defined as CC_50_/EC_50_.

## 3. Results

Among the 27 extracts tested only 16 plant species presented anti-HSV-1 activity. MNCC, EC_50_, and virucidal activity values obtained for each extract are showed in Table 1. No antiviral activity was detected in the algae extracts. Extracts with anti-HSV-1 activity were also tested for virucidal activity. In this experiment 6 of them showed virucidal values ≥90% of PFU reduction. With respect to antiviral activity and EC_50_ determination, *Psidium guajava, Phyllanthus niruri, and Limonium brasiliense* extracts had the highest values: 118, 60, and 185 *μ*g/mL, respectively. In addition, for the three extracts CC_50_ values were determined in mammalian cells and SI was calculated in each case. *P. niruri* presented the highest SI (42.37), followed by *P. guajava* (14.89). *L. brasiliense* showed an SI of 4.58. CC_50_ and SI values are shown in Table 2.

## 4. Discussion and Conclusions

The limited efficacy of the current treatment of HSV-1 infection enhances the need for novel therapies that include drugs with innovative viral targets and/or mechanisms of action. Since approximately 40% of modern drugs derived from natural sources and Ethnopharmacological knowledge has been an important source of information for the identification of bioactive compounds, we conducted this investigation with the aim to identify natural sources of compounds that could be potentially included in a formulation to be used in therapy against HSV-1.

 This work shows that ethanolic extracts of 16 plant species exhibit *in vitro* antiherpetic activity. The fact that 60% of all the plants assayed resulted positive demonstrates once again that traditional knowledge of medicinal plant usage is an efficient way of identifying biological and particularly antimicrobial activity. Half of the 16 positive plants showed EC_50_ values below 500 *μ*g/mL, about ten times greater than that of acyclovir, which is a purified compound. Among these, *P. niruri, Psidium incanum,* and *L. brasiliense* showed the highest anti-HSV-1 activity as was determined in plaque reduction assay.

 It is important to emphasize that the value obtained for *P. niruri *(EC_50_ = 60 *μ*g/mL) clearly correlates with its traditional use [13, 14]. In fact, this species is used to treat skin ulcers, sores, swelling, and itchiness of the skin [15] and other several illnesses including kidney and gall stones [9], jaundice, gonorrhea, and diabetes [16]. It also has been tested for antiviral activity against influenza [17], hepatitis B [18–20], and human immunodeficiency virus (HIV) [16]. Our results are in accordance with previous reports of antiherpetic activity of several species of the *Phyllanthus* genera [21–24]. With respect to *P. guajava* and *L. brasiliense*, there is no data about their antiherpetic activity. In fact, this is the first report demonstrating the inhibitory effect of HSV-1 replication *in vitro* of these plants. Remarkably, the three plant species have been widely used in traditional medicine by South American ancient populations for the treatment of several illnesses [13, 25].

 Since an antiviral compound should combine the highest effectiveness with the lowest cytotoxicity, it was important to evaluate the SI in order to determine the potential application of extract-derived compounds as antiviral agents. *P. niruri* has the highest SI resulting of a low CC_50_ value combined with a high affectivity.

*L. brasiliense* has an encouraging value of antiviral effectivity (EC_50_ = 185 *μ*g/mL), but the ethanolic extract of this plant is also highly toxic resulting in an SI of 4.58. Since ethanolic extracts are complex and heterogeneous and compounds of different chemical origin are present, it might be possible to separate toxic molecules from those responsible of the antiviral activity. For these, further studies with purification fractions of the extract should be carried out.

 On the other hand, it was important to differentiate direct viral particle inactivation from antiviral activity. In fact, virucidal activity might eclipse antiviral activity if tests of direct inactivation of viral particles are not performed. In our study, 6 plant extracts presented a positive activity (plaque reduction higher ≥ 90%). For this extracts one might assume that part of their activity relies on direct inactivation of the viral particles after virus adsorption and that this might also be occurring in the antiviral assay. However, this is not the case for extracts that combine low or no virucidal activity with positive antiviral activity (i.e., *P. niruri*, *P. incanum*, *Apium leptophyllum,* and *Conyza bonariensis*). For these anti-HSV-1 species another mechanism of action should exist since they act in postabsorption steps of the viral cycle.

 The results presented here justify further investigation of the extracts by bioguided fractionation procedures that hopefully will allow to identify active molecule(s) involved in anti-HSV-1 activity. If purified compounds with at least the antiviral values showed here are identified, this will turn them into candidates for *in vivo* trials and future antiherpetic formulations.

## Figures and Tables

**Table 1:** Antiviral and virucidal activity of each plant and algae species.

Species	MNCC^a^ (*μ*g/mL)	Antiviral activity	EC_50_^b^ (*μ*g/mL)	Virucidal activity (%)
Plants				
* Achyrocline satureioides*	2500	+	380	<90
* Apium leptophyllum*	5000	−	ND	ND
* Bauhinia candicans*	1250	+	1060	85
* Chenopodium ambrosioides*	2500	+	920	90
* Conyza bonariensis*	630	−	ND	ND
* Eryngium pandanifolium*	1250	−	ND	ND
* Heimia salicifolia*	1250	−	ND	ND
* Ibicella lutea*	1250	−	ND	ND
* Jaborosa runcinnata*	1250	−	ND	ND
* Lentinus edulis*	5000	−	ND	ND
* Limonium brasiliense*	500	+	185	99
* Margyricarpus pinnatus*	1250	+	320	95
* Phyllanthus niruri*	120	+	60	39
* Polygonum punctatum*	1250	+	1230	<90
* Psidium incanum*	250	+	118	20
* Psidium luridum*	310	+	1500	88
* Schinus molle*	630	+	50	<90
* Salvia guaranitica*	250	−	ND	ND
* Sesbania punicea*	1250	+	1100	<90
* Smilax gracilis*	2500	+	470	90
* Sommerfeltia spinulosa*	1250	−	ND	ND
* Tillandsia aeranthos*	2500	+	530	93
* Tillandsia usneoides*	1250	+	470	91
* Xanthium spinosum*	1250	−	ND	ND
Algae				
* Corallina officinalis*	630	−	ND	ND
* Chondria atropurpurea*	1250	−	ND	ND
* Grateloupia filicina*	2500	−	ND	ND
* Hypnea musciformis*	630	−	ND	ND
Acyclovir	120	+	45	99

^a^Maximum noncytotoxic concentration.

^b^Effective concentration 50%.

N/D: not determined.

+ Positive antiviral activity in the screening assay.

**Table 2:** CC_50_ and SI calculated for each anti-HSV-1 extract.

Plant species	CC_50_^a^ (*μ*g/mL)	SI^b^
*Psidium incanum*	1756	14.89
*Phyllanthus niruri*	2542	42.37
*Limonium brasiliense*	848	4.58
Acyclovir	3190	70.9

^a^Cytotoxic concentration 50%.

^b^Selectivity index. Ratio CC_50_/EC_50_.
